# MULTIVALENT ACE2 ENGAGEMENT ACTIVATES PIEZO1–SPHINGOMYELINASE SIGNALING IN SARS-COV-2 SPIKE-MEDIATED CELL–CELL FUSION

**DOI:** 10.64898/2026.10.22.761047

**Published:** 2026-10-23

**Authors:** Elena M. Zaitseva, Eugene N. Zaitsev, Kamran Melikov, Leonid V. Chernomordik

## Abstract

SARS-CoV-2 Spike not only fuses the viral envelope with host membranes to deliver viral RNA but also drives syncytium formation, a process that may contribute to viral spread and pulmonary pathology. Although the conformational transitions in Spike triggered by ACE2 engagement are well characterized, the host-cell signaling events initiated by Spike–ACE2 interactions remain less understood. Here, we examine how Spike activates acid sphingomyelinase (ASM) in ACE2-expressing A549 cells and how this activity promotes cell–cell fusion. Using ASM-deficient and complemented cells, exogenous ceramide, pharmacological perturbations, and a split-NeonGreen fusion assay, we support a fusion-promoting pathway in which Spike-induced ACE2 clustering activates the mechanosensitive ion channel PIEZO1, triggering lysosomal exocytosis. Secreted ASM, together with intracellular neutral sphingomyelinase, generates ceramide that enhances syncytium formation, likely through activation of protein phosphatases PP1 and PP2A. Our results suggest that these phosphatases promote fusion by facilitating actin cortex detachment and reducing actomyosin tension through dephosphorylation of ERM proteins. Our findings highlight dynamic changes in membrane lipid composition and membrane–cytoskeleton interactions as regulators of fusion and signaling in SARS-CoV-2 infection and, potentially, other viral and cellular systems.

## Full Text Availability

The license terms selected by the author(s) for this preprint version do not permit archiving in PMC. The full text is available from the preprint server.

